# Reproductive Performance of Hycole Rabbit Does, Growth of Kits and Milk Chemical Composition during Nine Consecutive Lactations under Extensive Rhythm

**DOI:** 10.3390/ani11092608

**Published:** 2021-09-05

**Authors:** Agnieszka Ludwiczak, Joanna Składanowska-Baryza, Beata Kuczyńska, Ewa Sell-Kubiak, Marek Stanisz

**Affiliations:** 1 Department of Animal Breeding and Product Quality Assessment, Poznań University of Life Sciences, Słoneczna 1, 62-002 Suchy Las, Poland; Joanna.skladanowska-baryza@up.poznan.pl (J.S.-B.); marek.stanisz@up.poznan.pl (M.S.); 2 Animal Breeding Department, Faculty of Animal Breeding, Bioengineering and Conservation, Warsaw University of Life Sciences, Ciszewskiego 8, 02-786 Warsaw, Poland; beata_kuczynska@sggw.pl; 3 Department of Genetics and Animal Breeding, Poznań University of Life Sciences, Wołyńska 33, 60-637 Poznań, Poland; ewa.sell-kubiak@puls.edu.pl

**Keywords:** rabbits production, extensive farming, kits growth, milk

## Abstract

**Simple Summary:**

The reproductive potential and longevity of rabbit does are the real determinants of the economic profitability of rabbit production. Another important characteristic is the quality of rabbit’s milk, as it determines the survival and growth of rabbit kits. The goal of this study was to examine the effect of parity order on the milk quality and the reproductive performance of Hycole does housed under intensive conditions of a commercial rabbit farm. The results of this trial and its duration allowed to trace the reproductive performance of Hycole females throughout their lifespan and to indicate the age boundary of a profitable reproductive performance of Hycole does.

**Abstract:**

The goal of this study was to analyze the reproductive performance of does, growth of their kits, and chemical composition of their milk over nine consecutive parities in order to indicate the boundary of female reproductive profitability. The novelty of this study results from the combinations of three factors: extensive reproductive rhythm, commercial farming conditions, and a period of nine consecutive parities, showing the actual lifespan of a rabbit doe on commercial farms. The data was collected on 60 Hycole females kept at a commercial rabbit farm. Throughout the study, 32 does were excluded due to different reasons (e.g., excluded by means of selection—43.8% and mortalities—25.0%). The does were first inseminated at 28 weeks of age. Following artificial inseminations were conducted 14–15 days after each parturition. All kits were weaned at the age of 35 days. The following characteristics were analysed: body weight of rabbit does at artificial insemination, milk production per lactation, litter size, litter weight, average kit weight, and milk chemical composition. Rabbit does had a significant decrease in kindling rate between the eighth and the ninth parity (by 10.0 percentage points; *p* = 0.039). The litter size at weaning in the ninth parity was significantly lower to litters weaned at other analysed parities. The amount of milk produced per lactation was affected by the parity order (6.31–6.76 kg; *p* = 0.042). The litter weights on day 21 and 35 were the lowest at ninth parity. The content of total solids (TS), solids-not-fat, and fat was affected by the parity order on both analysed lactation days. The content of TS and fat in rabbit milk was characterized with a decreasing trend over the analysed period, on both lactation days. The results clearly indicate that rabbit does under extensive reproductive cycles characterize with a very good reproductive performance and can be successfully used for reproduction even up to the eighth parity. However, further research is needed if keeping them longer will not be profitable.

## 1. Introduction

The reproductive longevity of rabbit does is listed among the most important traits of parental lines of rabbits and is determined by health and high reproductive performance [1]. Even though there is a common assumption that rabbit does are commercially used for reproduction for a maximum of 8–10 reproductive cycles, only a few studies support this decision statistically [2]. This comes as no surprise since most research on reproduction in rabbits presents results for two to three reproductive cycles, which are only a small part of their reproductive life [3,4,5]. Moreover, the vitality and growth of kits during the lactation period is dependent on the milk production and promoted by the unique chemical composition of rabbit milk. The health, growth rate, and survival of rabbit kits during the nursing period determines the litter size and litter weight at weaning, therefore, the fundamental understanding of the role of rabbit milk in the development of kits is needed. Based on the above, the thorough evaluation of does performance over their lifetime should covers multiple parities and combines the analysis of female reproduction traits with kits growth and milk quality.

The goal of the study was to trace the changes in the reproductive performance of does kept under an extensive reproductive cycle, as well as growth of their kits and chemical composition of their milk. The novelty of this study results from the combinations of three factors: extensive reproductive rhythm, commercial farming conditions, and a period of nine consecutive parities, showing the actual lifespan of a rabbit doe on commercial farms. Another unique aspect of this study is examination of rabbit milk chemical composition over nine consecutive parities.

## 2. Materials and Methods

### 2.1. Animals and Housing Conditions

The study was conducted on a commercial rabbit farm located in the western Poland. The building was equipped with industrial facilities (mechanical ventilation and conventional industrial furnace) allowing to maintain the uniform microclimate conditions all year round (temperature 16–20 °C, humidity 60–75%, and lighting schedule of 16 h light/8 h darkness). The reproductive performance of parental Hycole does was evaluated up to the ninth parity. The females were obtained from crossbreeding grandparental bucks (GPC) with grandparental does (GPD) [6]. At the start of the study, a total of 60 females was included. Rabbit does and their kits were kept in conventional wire-mesh cages including a feeder, nipple drinker, and a closable nest box. A single cage was 40 cm × 85 cm × 35 cm (width × length × height). Each cage was connected to a nest with dimensions of 30 cm × 45 cm × 30 cm. Throughout the study period, rabbit does were fed ad libitum with a commercial pelleted feed. The females and their kits had unlimited access to fresh water. First artificial insemination (AI) of nulliparous does was conducted at 28 weeks of age. This rather late age of first AI was the decision of the farm owner, who based it on observed improved reproductive performance. The farm was managed under a single-batch system. Artificial insemination was repeated each 45 days to inseminate the does 14–15 days after each parturition. The effectiveness of AI was checked by palpation performed 10 days after the procedure. In the case of ineffective AI, does were excluded from the experiment. Also the mortalities and other reasons to exclude the females from the study were recorded (i.e., small litter size, insufficient maternal capacities, severe mastitis). After birth, the litters were standardized by cross-fostering to obtain 10 kits per nest. From the moment of standardization until insemination, the nests were opened only once a day for about 10 min. After AI, the does had unlimited access to the does, drinker, and feeder.

### 2.2. Reproductive Performance Characteristics

The reproductive performance during the nine consecutive parities was assessed. The collected and analysed traits were: the live body weight of rabbit does at AI, kindling rate (the percentage of does kindling related to the number of inseminated does), litter size (at birth, and after standardization, on days: 14, 21, and 35 of lactation), and litter weight. The other trait analysed in this study was milk production per lactation (MP) calculated according to the equation developed by De Blas et al. [7]:

MP (kg) = 0.75 + 1.75 × W21 (kg)(1)

MP—milk production

LW21—litter weight at 21 day of lactation

### 2.3. Rabbit Milk Recovery and Examination

The rabbit milk recovery and quality examination were conducted using the methodology described in the study of Ludwiczak et al. [8]. The samples were collected during each of the nine analysed parities, on the 2nd and 21st day post-partum. A quantity of 12 mL of milk per rabbit doe was obtained into a plastic probe by gently massaging the mammary glands, and chilled to 4 °C directly after recovery. The milk was collected from randomly selected 25 females. Samples were transported under chilled conditions for examination of basic chemical composition (total solids, solids-not-fat, fat, protein, casein, lactose, and ash). The chemical composition of milk was determined by automated infrared analysis with a MilkoScan FT 120 analyzer (Foss Electric, Warsaw, Poland).

### 2.4. Statistical Analysis

All statistical analysis performed in this study were done with the SAS software package ver. 9.4 [9].

The PROC MIXED was applied to estimate the effect of the parity order on the body weight of rabbit does, litter weight, milk production and milk chemical composition (model 1), effect of the parity order on the litter size (model 2), as well as the effect of lactation day on the milk chemical composition (model 3). For this, the following three models were used:

Y_ijkl_ = μ + α_i_ + β_j_ + d_k_ + e_ijkl_ (model 1) (2)

where:

μ—the overall mean of the analysed trait,

α_i_—the fixed effect of the parity (i = 1, 2, …, 9),

β_j_—the fixed effect of litter size (l = 4, …, 15),

d_k_—random effect of the female (j = 1, 2, …, 60),

e_ijkl_—random error.

Y_ijk_ = μ + α_i_ + d_j_ + e_ijk_ (model 2) (3)

where:

μ—the overall mean of the analysed trait,

α_i_—the fixed effect of the parity (i = 1, …, 9),

d_j_—random effect of the female (j = 1, …, 60),

e_ijk_—random error.

Y_ijkl_ = μ + γ_i_ + β_j_ + d_k_ + e_ijkl_ (model 3) (4)

where:

μ—the overall mean of the analysed trait,

γ_i_—the fixed effect of day of lactation (i = 2, 21),

β_j_—the fixed effect of litter size (l = 4, …, 15),

d_k_—random effect of the female (j = 1, …, 60),

e_ijkl_—random error.

The random effect of a female was included in each model to correct for repeated observations per doe. Litter size as a fixed effect was added to the model to correct for the number of rabbits in the litter at the time of data collection. The “parity order*lactation day” interaction was initially also included in the model for the milk characteristics, but it was not statistically significant.

The kindling rate and the percentage of rabbit does excluded from the study was compared by χ^2^ test (PROC FREQ procedure in SAS). Tukey–Kramer adjustment was implemented for multiple comparisons of Least Squares Mean (LSM) differences, which was included as an additional analysis next to the models presented above.

## 3. Results

### 3.1. Reproductive Performance of Hycole Does and the Growth of Rabbit Kits

In Table 1 are presented all reasons for excluding the does and the percentage of animals affected by it. The fertility problem was the most often reason to exclude a female (43.8% of all cases), whereas the second most common reason was mortality during lactation (25% of all cases). The fact that does were removed from the farm by means of selection strategy allowed to maintain a high kindling rate up to the eight parity. High fertility was also promoted by controlled nursing. The amount of milk produced per lactation ranged from 6.31 kg to 6.76 kg, and was higher for the third and sixth parity compared to the first, second, seventh, eighth, and ninth parity. The reproductive performance of Hycole does examined in our study is presented in Table 2. The body weight of does at AI, kindling rate, and litter size varied between parities. The body weight of does was highly affected by the parity order (*p* = 0.001). The lowest body weight was observed at first parity (4.67 kg), whereas from the first to the fourth parity the body weight gradually increased. The total recorded difference in the body weight of females between first and fourth parity reached 8.5% increase. From the seventh parity till the end of the experiment this trait was almost stable. All rabbit does were characterized by a high kindling rate throughout the examined period. A clear decrease of this trait could only be observed between the eighth and the ninth parity (by 10.0 percentage points). The slightly significant effect of the parity order on the kindling rate (*p* = 0.039) can be explained by the fact that all the females with serious fertility problems (i.e., low litter size, severe mastitis, sore hocks or poor body condition) were excluded over the course of the study.

We also observed that the total born kits, born alive, and litter size on 21st and 35th lactation day significantly varied between consecutive parities (Table 3). The number of total born and born alive kits was the lowest at first and ninth parity (11.6 and 11.4 kits.) No significant differences were recorded on day 14 of lactation. The differences between parities recorded in litter size observed on days 21 and 35 were caused by kits mortality, which was significantly affected by the parity order. Litter weight at birth, on days 14, 21, and 35 of lactation significantly varied between the consecutive parities (Table 4). While the average kit weight was affected by the parity order only on day 35 post-partum (*p* = 0.001). The average kit weight recorded on day 35 of eighth and ninth parity showed a tendency to be lower compared to other examined parities.

### 3.2. Chemical Composition of Rabbit Milk

**Figure 1:**
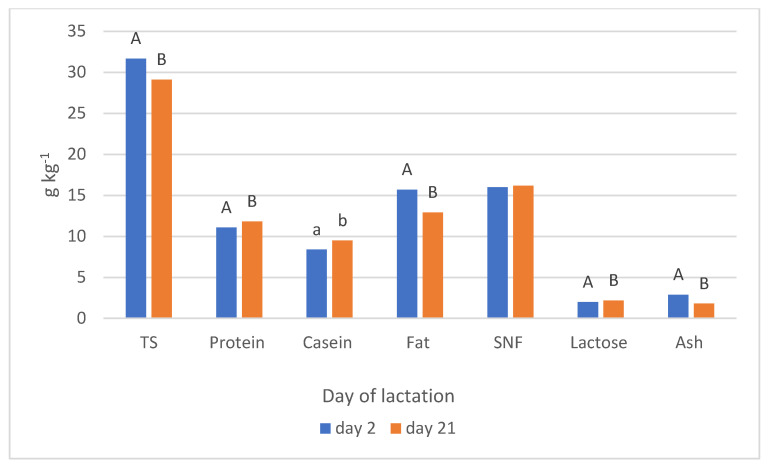
Effect of lactation day on rabbit milk chemical composition (TS—total solids; SNF—solids—not—fat); ^A, B (a, b)^ Values with different superscripts differ significantly at *p* < 0.01 (*p* < 0.05).

The effect of parity order on chemical composition of rabbit milk collected on day 2 and 21 is given in Table 5 and Table 6. On day 2 of lactations, the parity order caused a variation in the level of total solids (*p* = 0.001), SNF (*p* = 0.012), and fat (*p* = 0.039) in rabbit milk. The TS at first parity was much higher compared to parities eight and nine, by 2.36%. SNF showed the lowest values at the last two parities, and a decreasing trend over the analysed period. On day 21 of lactations, the parity order affected the content of TS (*p* = 0.044), fat (*p* = 0.015), and SNF (*p* = 0.036). TS and fat decreased over the analysed period. Fat content was significantly lower at parities eight and nine compared to parities one to four. Obviously, the content of chemical compounds in milk was affected by the day of lactation (Figure 1). Milk collected on day 2 was characterized by a greater content of TS, fat, and ash, and a lower content of protein, casein and lactose, compared to milk from day 21.

## 4. Discussion

Hycole does characteristics given by the producer emphasize the high reproductive performance of these rabbits: age of reproductive maturity in the range of 17–19 weeks, 89% birth rate, 10.7 kits born alive/kindling, 9.3 kits weaned/kindling, and 4.8–5.0 kg adult weight [6]. We observed an even better reproductive performance of this synthetic line of rabbits, and there are a few factors that promoted these results. Most commercial farms, and therefore, studies performed on rabbit does under intensive farming conditions, present the intensive reproductive rhythm with AI performed up to the 11th day post-partum. In our study, the extensive reproductive rhythm was used with the late age of the first service and as a result, almost had adult body weight [10]. This unquestionably had a positive effect on the reproductive performance of does, which allowed them to maintain high reproductive performance throughout the analyzed period. This can be explained by the already mentioned late age at first service, as well as extensive reproductive rhythm, properly performed culling strategy, and controlled nursing. Eiben et al. [4] also observed the positive effect of controlled nursing on reproductive performance in rabbits with higher fertility rate compared to free nursing (85.5% vs. 71.1%; *p* < 0.05).

Moreover, the literature underlines the relation between reproductive performance and the condition of does. Rebollar et al. [11] stated that nulliparous does characterize with higher fertility compared to the multiparous does, as the latter are exposed to significant energy deficits. In many studies it is highlighted that the inability of young does to meet high energy requirements for pregnancy and lactation during the first litters leads to high culling rates [12,13]. According to Rosell and de la Fuente [14], the average culling age for breeding does is 14.9 months and 6 parities. The authors observed that the first three parities are characterised by the highest risk of culling and mortality. Low productivity was given among the major causes of culling, while mastitis, poor condition, or sore hocks were noted less often. Rosell and de la Fuente [15,16] point to respiratory tract disorders as the main cause of mortality (including rabbits euthanized due to respiratory problems). Although we have noted some mortalities among rabbit does at different stages of reproductive cycle, their reproductive performance was high. This allows to speculate about other reasons for these mortalities than poor condition and energy deficits.

We have noted a significant influence of the parity order on the rabbit does’ body weight measured at AI. According to the literature, the changes of doe body weight with consecutive parities may be related to the effect of parity order on the body energy deficit [17,18]. Rabbit does in our study produced over 6.0 kg of milk per lactation. Although the milk production was generally high despite the parity order, the effect of parity on this trait was also clearly marked. The level of milk production per lactation was previously analysed by De Blas et al. [7] and ranged from 5.73 to 6.06 kg in a 30 d lactation. Moreover, the kits of does with the highest milk production (6.06 kg) were characterised with the highest litter weight on day 21 and at weaning. In the study of Xiccato et al. [19], the reproductive performance of rabbit does was analysed over three consecutive parities. The authors observed that the milk production increased with parity order, from 4548 g at first parity to 5410 g at third parity (*p* < 0.001). If we considered only the first three parities, we could observe the same tendency compared to Xiccato et al. [19]. We have noted an increase in milk production between the first and third parity, by 7.3%. Because in our study the litter weigh was affected by litter size and mortality, the parities with highest milk production did not exactly overlap with the parities with the greatest litter weaning weights.

The decrease in litter size from litter standardization till weaning recorded in our study was much lower compared to data in the literature [10]. The authors, Whitney et al. [20] conducted a survey on causes of pre-weaning mortality among young rabbits and noted that 12.4% of rabbits on commercial farms die in the pre-weaning period. From the total number of mortalities (347 kits) registered in the period from 0 to 4 weeks, 32.0% were stillborn; 21.3% deaths were caused by maternal neglect, inanition or hypothermia; 21.0% deaths were connected with inadequate husbandry, culling or fostering; and 18.4% causes of pre-weaning death cases remained not diagnosed. According to Rashwan and Marai [21], the pre-weaning mortality can be reduced through selection for greatest resistance to diseases in rabbits and is strongly related to the milk yield of the doe. Therefore, all the factors that decrease the milk production will lead to increased pre-weaning mortality. Therefore, high litter size at weaning recorded in our study was a result of a group of factors, with major ones being good condition of does and high milk production.

Similarly to our results, Mikó et al. [22] found that the parity order affected all the evaluated reproductive traits of rabbit does, including does body weight at AI, litter size, and litter weight. Litter weight is a composite of the number and individual weight of kits. According to the literature, the parity order and physiological status of rabbit does are among the major factors deciding about the birth weight of kits. Research conducted by Parigi–Bini and Xiccato [23] showed that kits of multiparous does were even 10% heavier at birth compared to kits from primiparous does. Rommers et al. [24] highlighted the individual milk intake and the litter size as the major factors deciding about the pre-weaning growth of kits. The effect of parity on litter weight observed in our study on days 14, 21, and 35, was rather related with kits mortality and litter size reduction than with the kit average weight.

Our study is unique as it evaluates the milk composition and quality in rabbits over nine parities. The knowledge that was available so far on this topic is very insufficient as the previous studies covered only short time periods, i.e., two or three parities [3,5]. These research neither reflect the true pattern of rabbit milk composition changes with parity order nor the effect of milk quality on kits growth and mortality. Because of the aforementioned, we decided to discuss our results with data obtained from studies on other farmed animals. According to the existing knowledge on farmed dairy cattle, the content of chemical compounds in milk and parity order are in strong relation, and the content of chemical compounds in milk tends to decrease with the parity order [25,26]. Similar observations were made in our study, showing that the rabbit milk composition has the same direction of changes compared to milk of farmed dairy animals, although the lactation in rabbits lasts only 30 days. The effect of lactation day on the chemical composition of rabbit milk observed in our study is consistent with the available literature [5,8,27,28,29].

## 5. Conclusions

To conclude, Hycole showed fluctuations in fertility, litter size, mortality, litter weight, milk production, and milk chemical composition over nine consecutive parities. The observation that should draw most attention is the kindling rate that is clearly decreasing between the eighth and the ninth parity. This indicates a decrease in reproductive performance of rabbit does around their eighth to ninth reproductive cycle. Nevertheless, the Hycole used in this study had a very good reproductive performance and high level of milk production throughout their reproductive lifetime. These results are partially related to the late age of their first service and an implemented extensive reproductive rhythm.

## Figures and Tables

**Table 1:** The percentage of rabbit does excluded from the study for different reasons (total number of does excluded from the study n = 32, this number included the females discarded also at ninth parity).

Reason	Does Excluded from the Study
% mortalities at the time of parturition	12.5 ^A^
% females with two consecutive ineffective AI	9.4 ^A^
% mortalities at the time of lactation	25.0 ^AB^
% excluded by means of selection	43.8 ^B^
% mortalities after weaning and before the ongoing parturition	9.4 ^A^

^A, B^ Values within a column with different superscripts differ significantly at *p* < 0.01.

**Table 2:** Comparison of the reproductive performance of Hycole does during the consecutive reproductive cycles (mean ± SE) with overall significance level of parity order (*p*-value) and differences between the parities.

Item	Parity Order									*p*-Value
	1	2	3	4	5	6	7	8	9	
Does (n)	60	54	51	48	47	45	43	42	36	
BW (kg)	4.67 ± 0.06 ^A^	4.89 ± 0.06 ^B^	4.91 ± 0.06 ^B^	5.10 ± 0.06 ^C^	5.02 ± 0.06 ^BC^	4.99 ± 0.06 ^B^	5.10 ± 0.06 ^C^	5.02 ± 0.06 ^BC^	5.02 ± 0.06 ^BC^	0.001
Kindling rate (%)	93.3 ^a^	88.9 ^b^	92.2 ^a^	95.8 ^c^	93.6 ^a^	91.1 ^ab^	95.3 ^c^	92.9 ^a^	83.6 ^d^	0.039
Milk production (kg)	6.31 ± 0.18 ^A^	6.33 ± 0.18 ^A^	6.76 ± 0.18 ^B^	6.58 ± 0.18 ^C^	6.44 ± 0.17 ^A^	6.67 ± 0.18 ^B^	6.52 ± 0.18 ^C^	6.47 ± 0.17 ^AC^	6.42 ± 0.17 ^A^	0.042

^A, B, C (a, b, c)^ Values within a row with different superscripts differ significantly at *p* < 0.01 (*p* < 0.05); BW (kg)—body weight of rabbit does at the day of artificial insemination.

**Table 3:** Comparison of the litter size traits of Hycole does between the consecutive reproductive cycles (means ± s.e.) with overall significance level of parity order (*p*-value) and differences between the parities.

Trait	Parity Order									*p*-Value
	1	2	3	4	5	6	7	8	9	
Born kits (n)										
Total	11.6 ± 0.4 ^a^	11.9 ± 0.5 ^ab^	12.4 ± 0.5 ^ab^	12.3 ± 0.4 ^ab^	12.2 ± 0.5 ^ab^	12.3 ± 0.5 ^ab^	12.1 ± 0.4 ^ab^	12.5 ± 0.5 ^b^	11.4 ± 0.4 ^a^	0.047
Alive	11.2 ± 0.5 ^a^	11.5 ± 0.5 ^ab^	12.1 ± 0.5 ^b^	11.6 ± 0.4 ^ab^	11.9 ± 0.5 ^ab^	11.9 ± 0.5 ^ab^	11.8 ± 0.4 ^ab^	12.2 ± 0.5 ^b^	11.0 ± 0.4 ^a^	0.043
Dead	0.31 ± 0.11	0.38 ± 0.11	0.26 ± 0.10	0.25 ± 0.10	0.21 ± 0.09	0.29 ± 0.10	0.24 ± 0.10	0.25 ± 0.10	0.42 ± 0.11	0.159
Litter size (n)										
Day 14	9.5 ± 0.3	9.3 ± 0.3	9.8 ± 0.3	9.9 ± 0.4	9.7 ± 0.4	9.4 ± 0.4	9.8 ± 0.3	9.6 ± 0.3	9.2 ± 0.3	0.687
Day 21	9.2 ± 0.3 ^ab^	9.1 ± 0.3 ^ab^	9.7 ± 0.3 ^a^	9.6 ± 0.4 ^ab^	9.6 ± 0.4 ^ab^	9.3 ± 0.4 ^ab^	9.7 ± 0.3 ^a^	9.5 ± 0.3 ^ab^	8.9 ± 0.4 ^b^	0.042
Day 35	9.1 ± 0.3 ^ab^	8.9 ± 0.3 ^ab^	9.5 ± 0.3 ^a^	9.4 ± 0.4 ^a^	9.3 ± 0.3 ^a^	9.1 ± 0.3 ^ab^	9.4 ± 0.3 ^a^	9.3 ± 0.4 ^a^	8.5 ± 0.5 ^b^	0.031

^a, b^ Values within a same row with different superscripts differ significantly at *p* < 0.01 (*p* < 0.05).

**Table 4:** Effect of parity order on litters weights and average weight of kits produced by Hycole does (mean ± s.e.) with overall significance level of parity order (*p*-value) and differences between the parities.

Item	Parity Order									*p*-Value
	1	2	3	4	5	6	7	8	9	
Litter weight (g)										
At birth	829 ± 12 ^Aa^	842 ± 15 ^A^	894 ± 16 ^BC^	872 ± 15 ^ABb^	883 ± 16 ^B^	882 ± 17 ^B^	874 ± 16 ^AB^	902 ± 18 ^Bb^	812 ± 17 ^A^	0.001
SD ^1^ litter	771 ± 5	776 ± 6	781 ± 5	774 ± 6	779 ± 5	776 ± 5	780 ± 5	782 ± 5	772 ± 6	0.867
Day 14	2831 ± 43 ^Aa^	2729 ± 42 ^ABb^	3003 ± 42 ^Ca^	2997 ± 42 ^Cb^	2785 ± 43 ^A^	2916 ± 43 ^Cb^	2866 ± 44 ^Aa^	2869 ± 42 ^Aa^	2683 ± 43 ^B^	0.001
Day 21	3598 ± 52 ^A^	3587 ± 53 ^A^	3907 ± 54 ^B^	3799 ± 53 ^B^	3681 ± 53 ^AC^	3839 ± 52 ^B^	3750 ± 53 ^C^	3749 ± 53 ^C^	3462 ± 52 ^C^	0.001
Day 35	8503 ± 135 ^A^	8411 ± 112 ^Aa^	9139 ± 121 ^B^	9259 ± 119 ^B^	8743 ± 132 ^Ab^	8639 ± 129 ^A^	8781 ± 128 ^A^	8502 ± 138 ^A^	7732 ± 131 ^C^	0.001
Average kit weight (g)										
At birth	71.5 ± 5.7	70.8 ± 5.7	72.1 ± 5.6	70.9 ± 5.7	72.4 ± 5.6	71.9 ± 5.6	72.3 ± 5.7	72.4 ± 5.7	71.3 ± 5.8	0.591
SD ^1^ litter	77.1 ± 4.2	77.6 ± 4.2	78.1 ± 4.1	77.4 ± 4.1	77.9 ± 4.1	77.6 ± 4.1	78.0 ± 4.3	78.2 ± 4.1	77,2 ± 4.3	0.859
Day 14	298 ± 14	294 ± 14	306 ± 13	303 ± 13	288 ± 14	311 ± 14	293 ± 13	299 ± 14	292 ± 4.2	0.757
Day 21	392 ± 19	387 ± 19	404 ± 19	395 ± 19	384 ± 19	409 ± 18	387 ± 19	396 ± 18	389 ± 19	0.862
Day 35	935 ± 21 ^ABb^	946 ± 22 ^AB^	963 ± 20 ^ABac^	986 ± 20 ^Ac^	941 ± 21 ^AB^	950 ± 20 ^AB^	935 ± 21 ^ABb^	915 ± 21 ^Bb^	910 ± 22 ^Bb^	0.001

^A, B, C (a, b)^ Values within a same row with different superscripts differ significantly at *p* < 0.01 (*p* < 0.05); ^1^ Standardised litters were standardised to 10 kits per litter after each parturition.

**Table 5:** Effect of the parity order on the chemical composition of rabbit milk on day 2 of lactation (g kg^−1^) with overall significance level of parity order (*p*-value) and differences between the parities.

Trait	Parity Order									SEM	*p*-Value
	1	2	3	4	5	6	7	8	9		
Does (n)	25	23	22	19	19	18	16	15	15		
TS	31.34 ^A^	31.09 ^B^	31.03 ^B^	30.94 ^B^	30.82 ^BC^	30.74 ^C^	30.70 ^C^	30.59 ^D^	30.60 ^D^	0.29	0.001
Protein	11.24	11.14	11.12	11.04	11.05	11.02	11.09	11.05	10.98	0.14	0.638
Casein	8.42	8.39	8.34	8.39	8.43	8.42	8.33	8.44	8.39	0.21	0.758
Fat	15.95 ^a^	15.83 ^ab^	15.82 ^ab^	15.76 ^b^	15.68 ^b^	15.64 ^bc^	15.57 ^c^	15.55 ^c^	15.56 ^c^	0.16	0.039
SNF	15.39 ^a^	15.26 ^a^	15.23 ^a^	15.18 ^ab^	15.14 ^b^	15.11 ^b^	15.13 ^b^	15.04 ^c^	15.04 ^c^	0.15	0.012
Lactose	2.03	2.01	2.01	2.02	2.00	2.01	1.99	1.98	1.98	0.02	0.835
Ash	2.12	2.11	2.1	2.12	2.09	2.07	2.05	2.01	2.08	0.05	0.501

TS—total solids; SNF—solids—not—fat; SEM—standard error of mean; ^A, B, C, D (a, b)^ Values within the same row with different superscripts differ significantly at *p* < 0.01 (*p* < 0.05).

**Table 6:** Effect of the parity order on the chemical composition of rabbit milk collected on day 21 of lactation (g kg^−1^) with overall significance level of parity order (*p*-value) and differences between the parities.

Trait	Parity Order									SEM	*p*-Value
	1	2	3	4	5	6	7	8	9		
Does (n)	25	23	22	19	19	18	16	15	15		
TS	29.16 ^a^	29.12 ^a^	29.01 ^ab^	28.94 ^ab^	28.49 ^c^	28.57 ^b^	28.48 ^c^	28.49 ^c^	28.44 ^c^	0.29	0.044
Protein	12.19	12.15	12.04	11.98	11.97	11.95	12.05	12.00	12.03	0.15	0.438
Casein	9.72	9.65	9.60	9.59	9.58	9.52	9.53	9.54	9.58	0.19	0.139
Fat	13.19 ^a^	13.14 ^a^	13.12 ^a^	13.11 ^a^	12.83 ^b^	12.75 ^b^	12.76 ^b^	12.68 ^bc^	12.57 ^c^	0.22	0.015
SNF	15.97 ^a^	15.98 ^a^	15.91 ^a^	15.83 ^ab^	15.66 ^b^	15.82 ^ab^	15.72 ^ab^	15.81 ^ab^	15.87 ^ab^	0.19	0.036
Lactose	2.04	2.03	2.08	2.09	1.89	2.06	1.86	2.01	2.03	0.05	0.431
Ash	1.74	1.8	1.79	1.76	1.8	1.81	1.81	1.8	1.81	0.06	0.683

TS—total solids; SNF—solids—not—fat; SEM—standard error of mean; ^a, b, c^ Values within a row with different superscripts differ significantly at *p* < 0.05.

## Data Availability

The data presented in this study are available on request from the corresponding author.
